# Supplementary material for: Arterial carboxyhaemoglobin levels in children admitted to PICU: A retrospective observational study
Source: PLoS One. 2019 Mar 7;14(3):e0209452. doi: 10.1371/journal.pone.0209452 (PMC6405068; doi:10.1371/journal.pone.0209452)
Supplement: S1 File — Letter of approval from UCL GOS Institute of Child Health Joint Research and Development Office. (PDF) [file pone.0209452.s001.PDF]

UCL INSTITUTE OF CHILD HEALTH

Great Ormond Street Hospital for Children
NHS Foundation Trust

Joint Research and Development Office
Division of Research and Innovation

020 7905 2249
Research.Governance@gosh.nhs.uk

31/08/2017

Dear Samiran Ray,

**Project Title**	Carboxyhaemoglobin levels in children on paediatric and neonatal intensive care
**Protocol version**	NHS R&D Form
**Protocol date**	NHS R&D Form
**REC Reference**	Not Applicable (GAfREC)
**R&D Reference**	17BB36
**Sponsor**	Great Ormond Street Hospital for Children NHS Foundation Trust

Notification of Great Ormond Street Hospital NHS Permission

The research approval process for the above named study has been completed successfully. I am pleased to issue approval on behalf of Great Ormond Street Hospital for Children NHS Trust (GOSH) for the above study to proceed.

All research carried out within this Trust must be in accordance with the principles set out in the Research Governance Framework for Health and Social Care (April 2005, 2nd edition, Department of Health (DoH)).

This approval is issued on the basis of the project documentation submitted to date.

This study is a Case Note Review, with access to previously collected, non-identifiable information/data under GAfREC 2011 and as a result is exempt from REC approval.

Failure to comply with the above conditions and regulations will result in the suspension of the research project.

Joint Research and Development Office
Division of Research and Innovation
UCL Institute of Child Health, 30 Guilford Street, London WC1N 1EH
Tel: 020 7905 2179 Fax: 020 7905 2201
www.gosh.nhs.uk

The child first and always

Please contact the Joint R&D Office if you require any further guidance or information on any matter mentioned above. We wish you every success in your research.

Yours sincerely,

Stephanie de Sa Marques Basset
Research Management and Governance Officer
Joint Research and Development Office

Joint Research and Development Office
Division of Research and Innovation
UCL Institute of Child Health, 30 Guilford Street, London WC1N 1EH
Tel: 020 7905 2179 Fax: 020 7905 2201
www.gosh.nhs.uk
